# Maternal physical activity affects yolk sac size and growth in early pregnancy, but girls and boys use different strategies

**DOI:** 10.1038/s41598-023-47536-4

**Published:** 2023-11-20

**Authors:** Alexander Vietheer, Torvid Kiserud, Cathrine Ebbing, Hemamaalini Rajkumar, Øystein Ariansen Haaland, Rolv Terje Lie, Roberto Romero, Jörg Kessler

**Affiliations:** 1 Department of Obstetrics and Gynecology, Haukeland University Hospital, Jonas Lies Vei 72, 5053 Bergen, Norway; 2 Maternal-Fetal-Neonatal-Research Western Norway, Department of Clinical Science, University of Bergen, Bergen, Norway; 3 Department of Global Public Health and Primary Care, University of Bergen, Bergen, Norway; 4 Centre for Fertility and Health, Norwegian Institute of Public Health, Oslo, Norway; 5 Pregnancy Research Branch, Eunice Kennedy Shriver National Institute of Child Health and Human Development, National Institutes of Health, U.S. Department of Health and Human Services, Bethesda, MD USA; 6 Department of Obstetrics and Gynecology, University of Michigan, Ann Arbor, MI USA; 7 Department of Epidemiology and Biostatistics, Michigan State University, East Lansing, MI USA

**Keywords:** Developmental biology, Embryology, Intrauterine growth, Organogenesis, Medical research, Environmental sciences, Environmental impact

## Abstract

This longitudinal study investigated the impact of actigraphy-measured maternal physical activity on yolk sac size during early development. The yolk sac, a transient extraembryonic organ, plays a crucial role in embryonic development and is involved in metabolism, nutrition, growth, and hematopoiesis. Prospectively collected data from 190 healthy women indicated that their total daily physical activity, including both light and moderate-vigorous activity, was associated with yolk sac growth dynamics depending on embryonic sex and gestational age. Higher preconception maternal physical activity was linked to a larger yolk sac at 7 weeks (95% CI [0.02–0.13 mm]) and a smaller yolk sac at 10 weeks’ gestation (95% CI [− 0.18 to − 0.00]) in male embryos; in female embryos, the yolk sac size was increased at 10 weeks’ gestation (95% CI [0.06–0.26]) and was, on average, 24% larger than that in male embryos (95% CI [0.12–0.38]). Considering the pattern of other maternal effects on yolk sac size—e.g., body composition and sleep duration—we suggest that physiological yolk sac adaptations occur in short, sex-specific time windows and can be influenced by various maternal factors.

## Introduction

The ability of an embryo and fetus to adapt to the intrauterine environment, including maternal factors, is considered to diminish over time^1–3^. Even before conception, maternal health factors are relevant for optimal endometrial preparation^4^ and implantation^5^. In addition, the response to environmental and maternal factors can vary between the sexes as early as the moment of conception based on their specific genetic and epigenetic potential^2,6–8^.

The rapid stages during early gestational development might be accompanied by corresponding rapid shifts in sensitivity to maternal and environmental cues. More detailed insight into the effect of specific factors and the sequence of events may reveal mechanisms of interest beyond fundamental knowledge, such as public health measures and clinical management^1^.

**Figure 1:**
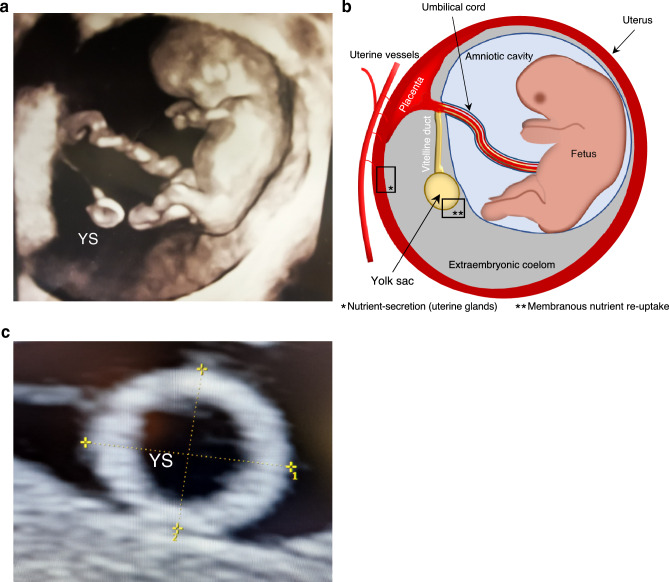
(**a**) 3D ultrasound of a 10-weeks’ embryo with a secondary yolk sac (YS). (**b**) Graphical illustration of the embryo-yolk sac connection with the yolk sac localized outside the amniotic cavity in the extraembryonic coelom. * Uterine glands secrete amino acids, ions, carbohydrates (glucose), lipids, proteins (e.g., cytokines, enzymes, hormones, growth factors, proteases and their inhibitors, and transporters)^61^. ** Yolk sac membrane with a vascular plexus envelope is involved in transport or resynthesis and exocytosis of nutrients, either directly into the surrounding blood vessels or the yolk sac cavity^11^. (**c**) Yolk sac size was assessed by two perpendicular outer-to-outer diameters measured thrice, and the mean was entered into the statistics.

The secondary yolk sac is a prominent structure during early human embryonic development that is easily visualized using ultrasound imaging during the first trimester. It is located in the exocoelomic cavity and remains connected to the developing embryo by the vitelline duct and its vessels (Fig. 1a,b). The yolk sac is involved in gastrointestinal tract formation, protein synthesis, stem cell production, and hematopoiesis^9–12^; for example, it is the origin of macrophage subtypes with high plasticity for epigenetic programming^13^. Through surface diffusion and transport proteins, the yolk sac membrane also facilitates gas exchange and provides nutrients until the placenta is sufficiently developed^9–12,14^.

Recently, our group found that in a healthy pregnancy, the yolk sac at approximately 8 weeks’ gestation is larger when the maternal height and weight are low, suggesting a compensatory adaptation to maintain embryonic growth within an optimal trajectory, and the effect was essentially observed in female embryos^15^. In a second study, a shorter maternal sleep duration was linked to a larger yolk sac at 7 weeks of gestation, but this was essentially limited to male embryos^16^.

This brings attention to another maternal factor, physical activity, which is related to healthy weight gain, improved maternal glucose control^17,18^, and favorable obstetric outcomes^18–22^. More specifically, maternal physical activity is associated with a larger placental volume, villous surface area and vascular volume^23^ and modulates factors related to placental angiogenesis^24,25^. Physical activity also downregulates genes involved in placental fatty acid and insulin transport, upregulates genes involved in amino acid transport across the placenta, and reduces oxidative stress^18,26^.

Based on this background, we speculate that physical activity in healthy women before and during early pregnancy affects the intrauterine environment. Thus, we hypothesize that these effects are reflected in the size of the yolk sac, which is involved in embryonic growth regulation, and that the effects are sex specific.

## Results

The cohort consisted of 436 eligible participants (Fig. 3 and Table 1), of whom 190 (43.6%) became pregnant and provided sufficient data for inclusion in the present study (all study data with keys are supplied in Supplementary Tables S1 and S2). These 190 women had regular menstrual cycles with a median of 28 days (range 24–35 days, interquartile range (IQR) 1 day) and successful pregnancies resulting in live-born neonates with a median pregnancy length of 281 days (IQR 12 days) according to the date of the last menstrual period (LMP) or 278.5 days (IQR 11.8 days) based on the embryonic crown-rump length (CRL) in the first trimester^27^. Generally, the rate of pregnancy complications was low, e.g., gestational hypertension (3.2%), gestational diabetes (3.7%), preterm birth (3.2%), and a 5-min Apgar score less than seven (1.1%). The demographic characteristics of the study cohort are provided in Table 1.

**Table 1:** Descriptive statistics of the participants^16^ are presented as the mean, standard deviation (SD), range (Min, Max), and interquartile range (IQR).

*n* = 190	Frequency	Missing	Mean	SD	Min	Max	IQR
Age (years)		0	29.0	3.1	20.0	35.0	27–31
Height (cm)		0	167.7	6.2	149.0	185.0	164–172
Weight (kg)		0	64.7	8.3	47.1	89.8	58.9–71.2
BMI		0	23.0	2.6	17.8	29.9	21–24.8
Lean body mass (kg)		0	45.7	3.8	36.0	55.6	43.0–48
Body fat (%)		0	28.8	5.5	15.9	41.9	25–32.9
Cycle length (days)		0	28.5	1.7	24	35	28–29
Parity		0					
0	89 (46.8%)						
1	79 (41.6%)						
≥ 2	22 (11.6%)						
Training efforts*		0					
None	3 (1.6%)						
Effortless walking	46 (24.2%)						
< 3 times week^−1^	90 (47.4%)						
≥ 3 times week^−1^	51 (26.8%)						

*Training efforts established based on a unvalidated questionnaire completed at study entry by each participant.

### Daily physical activity duration

In total, *92*.1% of all recorded days before conception and 93.7% of the recorded days at the end of the first trimester fulfilled our eligibility criteria^16^, and no participant was excluded from the analysis. The total duration of actigraphy and the frequency of recorded data for weekend days before conception did not differ from those recorded after conception. With notable individual variation, the total daily activity duration was 5 h and 55 min before conception (95% CI [5 h 37 min–6 h 13 min], and the duration was 1 h 36 min shorter at the end of the first trimester (95% CI [1 h 19 min–1 h 55 min]) (Table 2, Supplementary, Fig. S1). This pattern was similar for the different activity intensities (light and moderate-vigorous activity) (Table 2).

**Table 2:** Summary statistics of maternal physical activity based on actigraphy data of 190 low-risk pregnant women before pregnancy and at 13 weeks of gestation, presented with the number of measurements (*n*), mean or median, standard deviation (SD) or interquartile range (IQR), and 95% confidence interval of the mean (95% CI).

Term	*n*	Mean/Median	SD/IQR	95% CI
1st actigraphy recording (before pregnancy)	176			
Days before estimated conception*		36**	10–75**	(43.8–59.5)
Number of recorded days		3.7	0.7	(3.6–3.8)
Total activity (min day^−1^)		354.7	120.9	(336.7–372.7)
Light activity (min day^−1^)		259.7	94.1	(245.7–273.7)
Moderate or vigorous activity (min day^−1^)		96.8	55.9	(88.5–105.1)
2nd actigraphy recording (week 13)	178			
Gestational age (weeks)		13.2	0.8	(13.1–13.3)
Number of recorded days		3.7	0.6	(3.6–3.8)
Total activity (min day^−1^)		254.5	101.6	(239.5–269.5)
Light activity (min day^−1^)		198.8	83.1	(186.5–211.1)
Moderate or vigorous activity (min day^−1^)		57.5	36.2	(52.1–62.8)

Daily maternal physical activity duration was classified as total, light, and moderate or vigorous activity.

*Time to conception was calculated as the number of days from the start of the maternal actigraphy recording at inclusion to day 14 of the cycle that led to conception.

**The median or IQR was calculated.

### Yolk sac size

**Figure 2:**
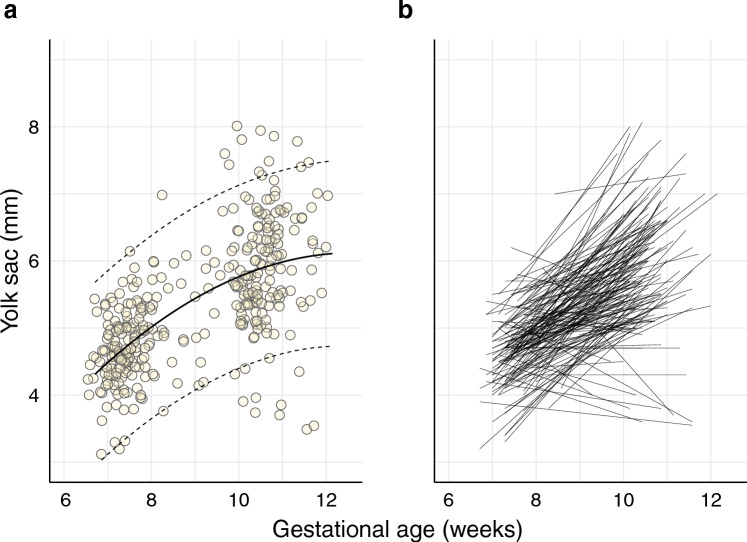
First and second yolk sac measurements by gestational age based on the last menstrual period. (**a**) In total, 358 measurements included the mean and 95% prediction band. (**b**) A line between the first and second yolk sac measurements representing the individual yolk sac growth rate. Line plot demonstrating the variations in yolk sac growth between the two measurements (no line is shown when only one measurement was available, and one participant was not included in this plot as the menstrual age at the time of the second measurement was an outlier beyond 14 gestational weeks).

A total of 358 yolk sac measurements were obtained through sonographic assessment at gestational weeks 7 and 10, showing an increase in the mean size from 4.7 mm at week 7 (95% CI [4.7–4.8]) to 5.9 mm at week 10 (95% CI [5.8–6.1]) (Fig. 2a, Table 3); the difference of 1.14 mm was significant (95% CI [0.99–1.29]). However, individual growth rates displayed considerable variation (Fig. 2b).

**Table 3:** Summary statistics of the ultrasound data of 190 low-risk pregnant women presented with subgroups (males and females), the number of measurements (*n*), mean, standard deviation (SD), 95% confidence interval of the mean (95% CI), and *p*-value (*p*) for the between-group tests.

Term	Sex	*n*	Mean	SD	95% CI	p*
**Week 7:** 1st measurement; GA (weeks)		180	7.6	0.7	(7.5–7.7)	
	♂	89	7.5	0.5	(7.4–7.6)	0.08
	♀	91	7.7	0.9	(7.5–7.8)	
**Week 7:** 1st yolk sac diameter (mm)		180	4.7	0.6	(4.7–4.8)	
	♂	89	4.7	0.6	(4.6–4.8)	0.39
	♀	91	4.8	0.6	(4.7–4.9)	
**Week 10:** 2nd measurement; GA (weeks)		178	10.6	0.8	(10.5–10.7)	
	♂	87	10.6	0.7	(10.5–10.8)	0.65
	♀	91	10.7	0.9	(10.5–10.9)	
**Week 10:** 2nd yolk sac diameter (mm)		178	5.9	0.9	(5.8–6.0)	
	♂	87	5.9	0.8	(5.7–6.1)	0.99
	♀	91	5.9	1.0	(5.7–6.1)	
**Week 7–10:** Yolk sac growth rate (mm week^−1^)		170	0.38	0.33	(0.33–0.43)	
	♂	81	0.37	0.29	(0.30–0.43)	0.67
	♀	89	0.39	0.36	(0.32–0.46)	

Gestational age (GA) was based on the last menstrual period.

*The unpaired t test was performed for the yolk sac data, and the Mann‒Whitney U test (Wilcoxon rank-sum test) was performed for the gestational age data.

### Inter- and intraobserver variability of yolk sac sonographic measurements

The reproducibility study of the yolk sac ultrasound measurements showed intraobserver variability of 0.08% and interobserver variability of 0.09%, corresponding to an intraobserver standard error of measurement (SEM) of 0.029 mm, with a 95% confidence interval (CI) of ± 0.056 mm. The interobserver SEM was 0.03 mm, resulting in a minimum detectable difference of 0.08 mm for the applied measurement technique.

### Effect of daily maternal physical activity duration on yolk sac size

When gestational age (GA) and embryonic sex were not accounted for, physical activity had no significant effect on yolk sac size, i.e., neither before conception nor at the end of the first trimester (0.03 mm·h^−1^, 95% CI [− 0.02 to 0.08]; and 0.00 mm·h^−1^, 95% CI [− 0.06 to 0.06], respectively).

### The effect of daily physical activity on yolk sac size is a function of fetal sex and GA

**Figure 3:**
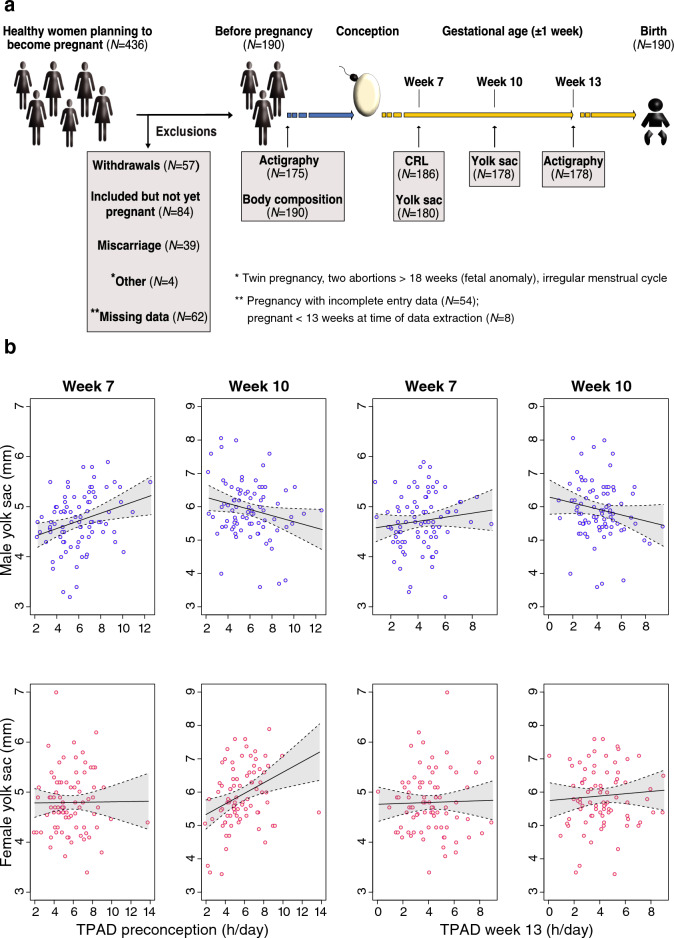
(**a**) Study protocol including the study population, participant exclusions, and study visits with the type and number of successful measurements. (**b**) Effect of the total daily activity duration (TPAD) before pregnancy (left half) and at gestational week 13 (right half) on the yolk sac size at 7 weeks and 10 weeks. A regression line and its 95% confidence interval are presented and grouped according to embryonic sex (males (blue) and females (red)).

**Figure 4:**
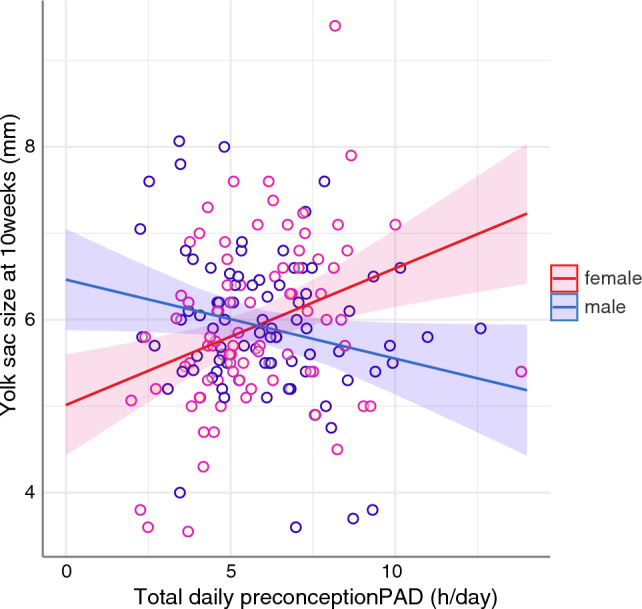
Yolk sac size of male and female embryos at 10 weeks of gestation according to total daily maternal physical activity (PAD) before pregnancy. The presented regression lines and their 95% confidence intervals are grouped according to embryonic sex.

At 7 weeks gestation, the yolk sac diameter of male embryos was larger when the prepregnancy physical activity duration was longer (i.e., 10% larger for the average amount of daily physical activity before pregnancy; *p* < 0.01). At this stage, such an effect was not evident in female embryos (*p* = 0.93*)*. However, at 10 weeks’ gestation, maternal physical activity was associated with yolk sac size in both male and female embryos (Fig. 3, Supplementary Table S3). At this stage, the relation between yolk sac size and maternal physical activity became negative for male embryos (*p* = 0.04), while for female embryos, a stronger and positive correlation was shown (*p* < 0.01), similar to the effect observed in male embryos at gestational week 7. Notably, the interaction between embryonic sex and daily maternal physical activity was also highly significant (0.24 mm·h^−1^; 95% CI [0.12–0.38]), underscoring the differential effect of maternal physical activity on yolk sac size at 10 weeks’ gestation, depending on the sex of the embryo (Fig. 4, Supplementary Table S3). When considering the average duration of maternal activity prior to conception, this effect translates to a yolk sac that is 24% larger in female embryos than in male embryos.

For the physical activity recordings at 13 weeks’ gestation, a similar relation between maternal physical activity and yolk sac size in male and female embryos at either 7- or 10-weeks’ gestation was observed but did not reach significance (*p* ≥ 0.1) (Supplementary Table S3; Fig. 3).

Adjusting the analysis for maternal age, parity, and body composition had a negligible impact on the results (Supplementary Tables S4–S7); the same applied to adjustments for GA (Supplementary Tables S4–S7), GA-adjusted yolk sac Z scores (Supplementary Table S8, Eq. 1 and Code C1), and quantile regression results (Supplementary Figs. S2–S5).

In a subanalysis, we stratified by time of inclusion due to the long study period and did not observe any significant effect on our results (Supplementary Tables S4–S7). The same applied for the few women with pregnancy complications in the study; their exclusion from the analysis did not alter our results (Supplementary Tables S4–S7).

### Effect of maternal physical activity on yolk sac growth velocity (mm·week^−1^)

In addition to the association between daily maternal physical activity duration and yolk sac size at different GAs (weeks 7 and 10), we also found an effect on yolk sac growth dynamics. In both male and female embryos, the recorded maternal physical activity before pregnancy was associated with variation in yolk sac growth velocity (mm·week^−1^) but differed by 10% per hour of physical activity between the sexes, and this was highly significant (*p* < 0.01). In contrast to male embryos, where yolk sac growth was lower at higher activity durations (*p* < 0.01), female embryos showed higher growth between 7 and 10 weeks’ gestation (*p* = 0.01) (Supplementary Table S9).

### Effect of maternal physical activity intensity on yolk sac size

**Figure 5:**
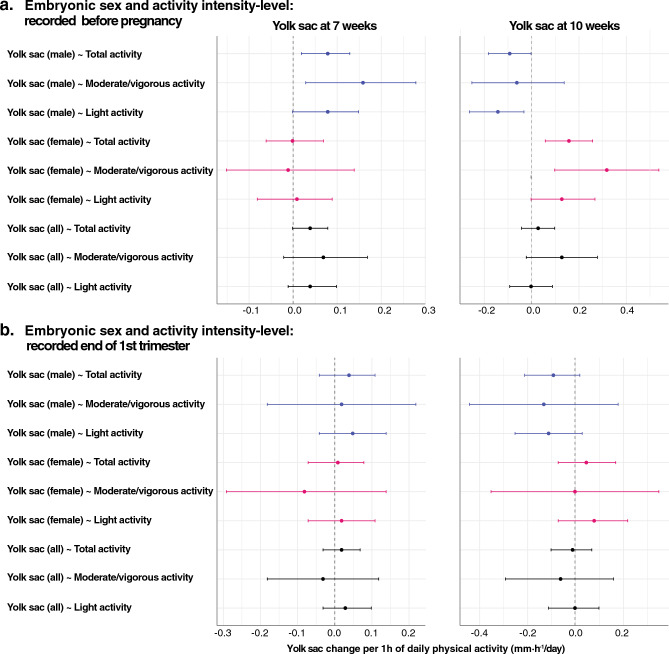
Forest plot showing the effect of physical activity intensity on yolk sac size according to embryonic sex and the time of the actigraphy recording: (**a**) before pregnancy and (**b**) at the end of the 1st trimester. Coefficients are presented with 95% confidence intervals.

The total daily duration of maternal physical activity was classified based on intensity, as light versus moderate-vigorous physical activity. Even within these subcategories, the impact of maternal physical activity, dependent on sex and GA, was similar to that observed for the total physical activity duration (Fig. 5).

## Discussion

This study of low-risk human pregnancies demonstrated that maternal physical activity before and during early pregnancy affects embryonic development, in this case, yolk sac size. A graded yolk sac response based on maternal physical activity duration was observed across all activity levels, encompassing both light and moderate-vigorous activities. Notably, we found that the effect was sex dependent, with different time windows and directions of impact (Fig. 3b, 4). Additionally, the sex-specific effect on yolk sac growth rate between gestational weeks 7 and 10 revealed the dimension of an inverse effect on yolk sac growth dynamics for male and female embryos. Therefore, we hypothesize that high physical activity levels may strain the intrauterine environment and cause compensatory enlargement of the yolk sac surface at different GAs to ensure adequate nutritional support for embryonic growth, determined by embryonic sex.

**Figure 6:**
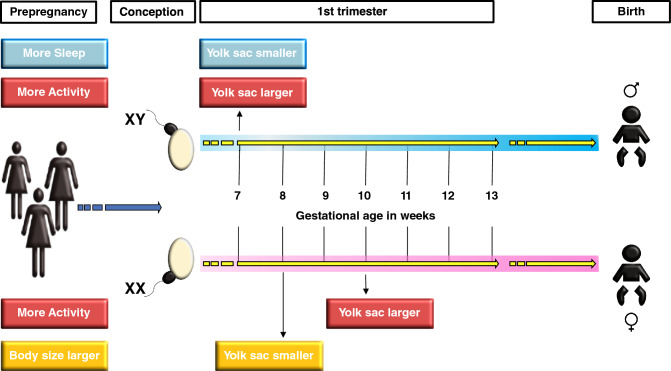
Effect of preconception maternal factors on yolk sac size: i.e., the effect of maternal sleep duration^16^, total maternal physical activity duration, and maternal body size (weight and height)^15^. The figure illustrates the sex and time-dependent effects during the 1st trimester. The time windows, where these effects can be observed, are short.

Based on the two previous studies^15,16^ and the present study, a distinct pattern emerges: sensitive windows in embryonic development seem short, and the timing and effects are sex-specific (confer overview in Fig. 6). For example, at 8 weeks of gestation, a larger yolk sac size in female embryos develops when the maternal height and weight are low^15^. On the other hand, at 7 weeks’ gestation, a larger yolk sac is seen in male embryos when the maternal sleep duration is short^16^. The present study showed that an extended maternal physical activity leads to a larger yolk sac in male embryos at 7 weeks’ gestation, while in female embryos, an extended physical activity is associated with a smaller yolk sac at 10 weeks’ gestation. This figure illustrates not only the sex-specific modifications of the observed effects in terms of timing and direction but also underscores the importance of precise and frequent observations—during a phase of rapid progression through consecutive developmental stages—to capture such effects. Animal studies have also provided some evidence supporting the effect of environmental factors, such as temperature, nutrition, and noise, on the yolk sac^28–31^, but yolk sac development and implantation mechanisms vary among species^5,10,11^.

Nevertheless, the findings of the present study support the concept of the compensatory enlargement of the yolk sac surface to ensure adequate nutritional support for embryonic growth^15^. In nonpregnant individuals, exercise is known to reduce visceral blood flow to meet the metabolic demand of the working muscles^32^. This reduction in visceral blood flow is accompanied by increased vasodilatation through the release of nitric oxide (NO)^33^ and endothelium-dependent hyperpolarization^34^. It has also been suggested that physical activity induces shear stress and intermittent fluctuations in substrate and oxygen delivery, resulting in hypoxic strain, which generates a repetitive stimulus triggering a feto-maternal response with increased placental vascularization^26,35^.

However, compared with the increase in yolk sac size, the association of physical activity and fetal body composition^36–39^, fetal growth, placental size^36,40–43^, and placental circulation^23^ are relatively late pregnancy responses to multiple factors and events. A significant feto-maternal connection via placental circulation is not established before twelve weeks of gestation^44^ and therefore is unlikely to explain variations in yolk sac size. Nevertheless, the underlying mechanisms may be similar because they both occur within the same organ, the uterus, with the same supplying vasculature, myometrium, and endometrium that includes glands surrounded by vessels. Therefore, it is plausible that fluctuations capable of influencing placental development may also influence histotrophic nutrition at earlier stages of pregnancy via the uterine glands and vasculature^10,11^. Furthermore, sex steroid levels, which are associated with physical activity in women^45^, are widely recognized to influence both the menstrual cycle and the timing of ovulation, as well as the composition of the endometrium and its glands.

The sex-specific response in size and growth dynamics shown in the present study is in line with other reports on sexual dimorphism in response to environmental factors during pregnancy in animal and in vitro studies^2^. We envisage that physical activity might act as a natural stress factor leading to the physiological adaptive response of the yolk sac (i.e., increasing size and thereby surface area that facilitates gas exchange and nutrient uptake during the period before the placenta is sufficiently developed).

### Study strengths

The strengths of this study lie in its prospective longitudinal design, which includes maternal data from the preconception period, and the inclusion of many healthy women who conceived naturally, without the confounding influence of hormonal treatments commonly used in assisted reproduction. Furthermore, the observed opposite effects on male and female embryos at 10 gestational weeks provide robust evidence for a sex-dependent effect of maternal physical activity on yolk sac size.

Additionally, the effect on yolk sac size at gestational weeks 7 and 10 is corroborated by the sex-specific effect on the yolk sac growth rate and strengthens the internal validity of the study.

Another strength is the utilization of alternative statistical models and quantile regression models, which consistently yielded the same results. This demonstrates that the results were not dependent on skewed data, systematic distribution differences, or extreme values.

To ensure the accuracy of yolk sac measurements, intra- and interobserver variability were calculated, confirming sufficient measurement precision, which was unbiased by any observer. Confounding observer effects are unlikely since maternal physical activity is not inherently related to the ultrasound procedure itself.

The ultrasound operators were blinded to embryonic sex, as embryonic sex was unknown at the time of observation.

Multiple regression analyses examining the association between maternal physical activity and yolk sac size did not reveal any significant observer effects (ultrasound operator) or effects of maternal age, parity, weight, height, BMI, lean body mass, body fat percentage, GA, time of inclusion, or the inclusion of the few participants with pregnancy complications.

### Study limitations

Due to the study design, we cannot be certain that the activity patterns recorded before pregnancy, although close to conception (Table 2), continued into early pregnancy. In addition, the study included only two yolk sac measurements (gestational weeks 7 and 10). Both factors imply that we cannot infer whether the variation in yolk sac observations stems from pre- or periconception variations in the intrauterine environment (i.e., the vasculature, myometrium, and endometrium with endometrial glands) or whether we observed an ongoing sex-specific impact of maternal physical activity during gestation within the specific time windows of gestational weeks 7 and 10.

An additional use of activity diaries or continuous measurement as well as more frequent yolk sac measurements would have strengthened the conclusions and provided deeper insight. However, the effect of different intensities of physical activity on the yolk sac could also be traced later in pregnancy (at 13 weeks’ gestation), suggesting that the physical activity pattern was similarly distributed in the population throughout the entire period.

Other challenges to control for are sex steroid levels, mental stress, and maternal nutrition. Stress causes a hormonal response similar to that of exercise, and nutrition is closely related to energy metabolism; thus, both might be confounders. The study population, however, consisted of healthy women with no history of chronic diseases or risk factors, and the chance of chronic psychological stress in this population should therefore be low. Confounding by differences in maternal nutrition also seems unlikely, as maternal body composition, which is closely related to energy metabolism and nutrition, did not significantly affect our results (Supplementary Tables S4–S7).

## Conclusion

Normal human embryonic development is sensitive to maternal cues. Here, we showed that maternal physical activity influences human yolk sac development in a graded fashion. Second, embryonic sex determines the timing, degree, growth dynamics, and direction of the effect. Third, the time frames for these effects seem to be rapidly changing, short phases at this stage of pregnancy.

## Methods

We studied the effects of maternal physical activity on the yolk sac in a prospective, longitudinal study of healthy nonsmoking women who planned to conceive naturally. The study is embedded in the ongoing CONIMPREG research program^16,46^.

### Data collection

During the period 2014–2020, women aged 20–35 years with a BMI of 18–30 kg/m^2^ were recruited through social media (targeted Facebook® advertisements) and posters, provided that they had an uncomplicated obstetric history, a regular menstrual cycle, did not use contraceptives during the month before study entry and had no chronic diseases or fertility problems. If the women did not conceive within six sampling cycles, they were excluded from the study.

The participants were assessed at four consecutive study visits (Fig. 3a). At the first visit—before conception—maternal height and body composition were measured, immediately followed by the first actigraphy recording. The second visit was scheduled—based on the first day of the LMP—at 7 ± 1 weeks’ gestation. At this time, we confirmed the viability of the embryo and the length of gestation^47^ and assessed the yolk sac. At the third visit (10 ± 1 weeks’ gestation), the yolk sac measurements were repeated, and finally, at the fourth visit (13 ± 1 weeks’ gestation), maternal body composition and activity duration were reassessed.

### Height, weight, and maternal body composition

Before conception, height was measured with a wall-mounted stadiometer^48^, and weight was measured digitally using bioelectrical impedance analysis (model BC-418, Tanita, Tokyo, Japan). The percentage of body fat was estimated using the instrument’s computer software, and lean body mass was calculated by subtracting body fat mass from total body weight. Measurements were carried out as recommended by the manufacturer^49^.

### Physical activity

Maternal physical activity was recorded before conception and at gestational week 13 using the SenseWear Mini Armband Actigraph (model MF-SW, BodyMedia, Pittsburgh, PA, USA). This wireless, noninvasive activity monitor incorporates triaxial accelerometry, heat flux, galvanic skin response, skin temperature, and near-body temperature measurements with a sampling frequency of 32 Hz. All information, plus information on sex, age, height, and weight, is considered in proprietary algorithms to predict physical activity at the level of 1.4 metabolic equivalents (METs)^50^. In accordance with the Sedentary Behavior Research Network (SBRN) consensus and American College of Sports Medicine (ASM) guidelines, the recordings were classified as light activity at ≥ 1.5 METs < 3.0, moderate at ≥ 3.0 METs < 6.0, and vigorous at ≥ 6.0 METs^51,52^. The monitor was worn on the upper posterior part of the nondominant arm for 4 days^53^, and the recording started at midnight. Raw data were processed and summarized using SenseWear Pro analysis software (SenseWear Professional, version 8.0.0.2903, Body Media) and exported into Excel workbooks (Microsoft Office, Excel version 2016, Redmond, WA, USA). Sampling days were excluded from the statistical analyses when data loss in a single day exceeded 6%. This or earlier versions of this actigraph have been validated for physical activity measurements^50,54,55^, including measurements during pregnancy^56,57^. The results of the included pregnant women were in good agreement with results from earlier versions of this monitor and other actigraphs or methods^46^.

### Embryonic measurements

At gestational weeks 7 and 10, ultrasound measurements were carried out by a group that consisted of seven obstetricians using a 6–12 MHz transvaginal transducer (Voluson Expert E8; GE Medical Systems, Kretz Ultrasound, Zipf, Austria). The transducer output power was set to be low, with a thermal index (TI) always below 1.0^58^. Viability of the embryo was ensured by employing clinical guideline safety criteria^59^, and the length of gestation was confirmed by the CRL^47^ determined as the mean of three measurements. The yolk sac size was determined as the average of two perpendicular outer diameters measured thrice^15^ (Fig. 1c).

### Inter- and intraobserver variability of the yolk sac measurements

To calculate the inter- and intraobserver variability of yolk sac size measurements, we expanded our study in 2023 by utilizing prospectively collected data (Supplementary Tables with keys in S10 and S11) from the same study cohort (CONIMPREG). Embryonic yolk sacs (*n* = 19) were assessed either at gestational week 7 or week 10, and video sequences (ultrasound loops) were generated and stored in the machine’s local archive.

All seven ultrasound operators were instructed to select the best yolk sac image from the sequence and measure the yolk sac using the previously described method. This involved measuring the perpendicular diameters three times and calculating the mean size. After a minimum of one day, the procedure was repeated to assess intraobserver variability (repeatability).

### Statistics

Statistical analysis was performed using R (Foundation for Statistical Computing, version 4.1, Vienna, Austria) and R-studio (Integrated development for R, Boston, MA, USA) software.

The mean and standard deviation (SD) with minimum and maximum values were calculated for each continuous variable, and frequencies and proportions were calculated for categorical variables. When the distribution was asymmetric, the median and IQR are reported. In addition, the 95% CIs of the mean were calculated for the recorded physical activity intensities, the number of days with recorded data, the frequency of physical activity on weekend days, and the CRL and yolk sac size with GA at the time of the measurements.

Ordinary least square linear (OLS) regression models were used to analyze the association of yolk sac size with maternal physical activity duration before pregnancy and at the end of the first trimester (week 13). Linearity assumptions and normal distribution of the residuals were ascertained. The regression models were fitted with and without embryonic sex stratification. In addition, we tested the effect of embryonic sex on the maternal physical activity-yolk sac relation by adding the interaction term (embryonic sex*maternal physical activity) to the OLS model. OLS regression results were compared with results from quantile regression, including iterated reweighted least squares regressions (Huber weights and bisquare weighting), and heteroskedastic methods (sandwich variance estimators). In the subanalysis of our main findings, we replaced the yolk sac diameter with the yolk sac Z score that was calculated employing multilevel growth models, accounting for repeated measurements and GA (Supplementary Equation EQ1 and code C1). In addition, we controlled for physical activity effects in the original OLS model for GA. Likewise, maternal age, parity, and body composition parameters (i.e., height, weight, body mass index, lean body mass, and body fat percent) were added one by one to the primary model and were included if they notably altered the effect size of the association. We also stratified by time of inclusion and assessed the effect of three equally sized time categories between 2014 and 2020 by adjusting the regression model for these strata. Finally, we performed regression analyses with and without participants who experienced complications or unfavorable obstetric outcomes (i.e., hypertensive complications, gestational diabetes, preterm birth, and a 5-min Apgar score less than seven). As measures of fit, the adjusted *R*-squared and Akaike information criterion were calculated. Differences between the regression models were tested using analysis of variance (ANOVA) methods. Differences in variables from the summary statistics were tested with unpaired and paired parametric or nonparametric tests.

The assessment of intra- and interobserver variability, along with the associated SEM (SEM-intraobserver and SEM-interobserver), as well as the minimum detectable difference, was conducted using a two-way ANOVA method, as outlined by Popović and Thomas^60^. The necessary variances were derived either directly or indirectly by utilizing the variances expressed as multiple squares for the various factors of the model (i.e., observer, subject, the interaction between the observer and subject) and the residual variation (Code is provided in Supplementary Code C2).

### Ethics declaration and consent

The study was approved by the Regional Committee for Medical Research Ethics Southeast Norway (REK Southeast, ref. 2013/856a). E-mail: rek-sorost@medisin.uio.no. Written informed consent was obtained from all participants, and all research was performed in accordance with relevant guidelines and regulations.

### Supplementary Information

Supplementary Information 1.

Supplementary Information 2.

Supplementary Table S1.

Supplementary Table S10.

## Data Availability

All data generated or analyzed during this study are included in this published article (and its supplementary information files).

## References

[1] Hanson MA, Gluckman PD (2014). Early developmental conditioning of later health and disease: physiology or pathophysiology. Physiol. Rev..

[2] Rosenfeld CS (2015). Sex-specific placental responses in fetal development. Endocrinology.

[3] Workalemahu T (2018). Genetic and environmental influences on fetal growth vary during sensitive periods in pregnancy. Sci. Rep..

[4] Ljubic A, Abazovic D, Ljubic D, Pirkovic A, Perovic A (2020). Induced Abortion and Spontaneous Early Pregnancy Loss—Focus on Management.

[5] Carter AM (2021). Unique aspects of human placentation. Int. J. Mol. Sci..

[6] Bermejo-Alvarez P, Rizos D, Lonergan P, Gutierrez-Adan A (2011). Transcriptional sexual dimorphism during preimplantation embryo development and its consequences for developmental competence and adult health and disease. Reproduction.

[7] Deegan DF, Engel N (2019). Sexual dimorphism in the age of genomics: How, when, where. Front. Cell Dev. Biol..

[8] Eriksson JG, Kajantie E, Osmond C, Thornburg K, Barker DJ (2010). Boys live dangerously in the womb. Am. J. Hum. Biol..

[9] Burton GJ, Hempstock J, Jauniaux E (2001). Nutrition of the human fetus during the first trimester—A review. Placenta.

[10] Burton GJ, Cindrova-Davies T, Turco MY (2020). Review: Histotrophic nutrition and the placental-endometrial dialogue during human early pregnancy. Placenta.

[11] Ross C, Boroviak TE (2020). Origin and function of the yolk sac in primate embryogenesis. Nat. Commun..

[12] Mäkikallio K, Tekay A, Jouppila P (1999). Yolk sac and umbilicoplacental hemodynamics during early human embryonic development. Ultrasound Obstet. Gynecol..

[13] Chen S, Yang J, Wei Y, Wei X (2020). Epigenetic regulation of macrophages: From homeostasis maintenance to host defense. Cell Mol. Immunol..

[14] Goh I (2023). Yolk sac cell atlas reveals multiorgan functions during human early development. Science.

[15] Odland Karlsen H (2018). The human yolk sac size reflects involvement in embryonic and fetal growth regulation. Acta Obstet. Gynecol. Scand..

[16] Vietheer A, Kiserud T, Lie RT, Haaland ØA, Kessler J (2022). Effect of maternal sleep on embryonic development. Sci. Rep..

[17] Ming WK (2018). The effect of exercise during pregnancy on gestational diabetes mellitus in normal-weight women: A systematic review and meta-analysis. BMC Pregnancy Childbirth.

[18] Reyes LM, Davenport MH (2018). Exercise as a therapeutic intervention to optimize fetal weight. Pharmacol. Res..

[19] Mudd LM, Owe KM, Mottola MF, Pivarnik JM (2013). Health benefits of physical activity during pregnancy: An international perspective. Med. Sci. Sports Exerc..

[20] Barakat R, Franco E, Perales M, López C, Mottola MF (2018). Exercise during pregnancy is associated with a shorter duration of labor. A randomized clinical trial. Eur. J. Obstet. Gynecol. Reprod. Biol..

[21] Witvrouwen I, Mannaerts D, Van Berendoncks AM, Jacquemyn Y, Van Craenenbroeck EM (2020). The effect of exercise training during pregnancy to improve maternal vascular health: Focus on gestational hypertensive disorders. Front. Physiol..

[22] Russo LM, Harvey MW, Pekow P, Chasan-Taber L (2019). Physical activity and risk of cesarean delivery in hispanic women. J. Phys. Act. Health.

[23] Jackson MR, Gott P, Lye SJ, Ritchie JW, Clapp JF (1995). The effects of maternal aerobic exercise on human placental development: Placental volumetric composition and surface areas. Placenta.

[24] Bhattacharjee J, Mohammad S, Goudreau AD, Adamo KB (2021). Physical activity differentially regulates VEGF, PlGF, and their receptors in the human placenta. Physiol. Rep..

[25] Hardy DB, Mu X, Marchiori KS, Mottola MF (2021). Exercise in pregnancy increases placental angiogenin without changes in oxidative or endoplasmic reticulum stress. Med. Sci. Sports Exerc..

[26] Ramírez-Vélez R, Bustamante J, Czerniczyniec A, Aguilar-de-Plata AC, Lores-Arnaiz S (2013). Effect of exercise training on eNOS expression, NO production and oxygen metabolism in human placenta. PLoS ONE.

[27] Robinson HP, Fleming JE (1975). A critical evaluation of sonar “crown-rump length” measurements. Br. J. Obstet. Gynaecol..

[28] Fiksen, Ø. & Folkvord, A. Maternal effects and the benefit of yolk supply in cod larvae in different environments—A simulation model. ICES Council Meeting, 1–6 (1999).

[29] Lara RA, Vasconcelos RO (2021). Impact of noise on development, physiological stress and behavioural patterns in larval zebrafish. Sci. Rep..

[30] Mikec M (2006). Influence of environmental and nutritional stressors on yolk sac utilization, development of chicken gastrointestinal system and its immune status. World’s Poultry Sci. J..

[31] Watkins AJ (2008). Adaptive responses by mouse early embryos to maternal diet protect fetal growth but predispose to adult onset disease. Biol. Reprod..

[32] Delp MD (1998). Differential effects of training on the control of skeletal muscle perfusion. Med. Sci. Sports Exerc..

[33] Schuler G, Adams V, Goto Y (2013). Role of exercise in the prevention of cardiovascular disease: Results, mechanisms, and new perspectives. Eur. Heart J..

[34] Gündüz F (2011). Exercise training enhances flow-mediated dilation in spontaneously hypertensive rats. Physiol. Res..

[35] Bergmann A, Zygmunt M, Clapp JF (2004). Running throughout pregnancy: Effect on placental villous vascular volume and cell proliferation. Placenta.

[36] Clapp JF (2002). Continuing regular exercise during pregnancy: effect of exercise volume on fetoplacental growth. Am. J. Obstet. Gynecol..

[37] Hopkins SA, Baldi JC, Cutfield WS, McCowan L, Hofman PL (2010). Exercise training in pregnancy reduces offspring size without changes in maternal insulin sensitivity. J. Clin. Endocrinol. Metab..

[38] Harrod CS (2014). Physical activity in pregnancy and neonatal body composition: the Healthy Start study. Obstet. Gynecol..

[39] Bisson M (2017). Influence of maternal physical activity on infant’s body composition. Pediatr. Obes..

[40] Clapp JF, Kim H, Burciu B, Lopez B (2000). Beginning regular exercise in early pregnancy: Effect on fetoplacental growth. Am. J. Obstet. Gynecol..

[41] Clapp JF (2006). Influence of endurance exercise and diet on human placental development and fetal growth. Placenta.

[42] Rodríguez I, González M (2014). Physiological mechanisms of vascular response induced by shear stress and effect of exercise in systemic and placental circulation. Front. Pharmacol..

[43] Hilde G, Eskild A, Owe KM, Bø K, Bjelland EK (2017). Exercise in pregnancy: an association with placental weight. Am. J. Obstet. Gynecol..

[44] Burton GJ, Jauniaux E (2018). Development of the human placenta and fetal heart: Synergic or independent. Front. Physiol..

[45] Ennour-Idrissi K, Maunsell E, Diorio C (2015). Effect of physical activity on sex hormones in women: A systematic review and meta-analysis of randomized controlled trials. Breast Cancer Res..

[46] Vietheer A, Kiserud T, Lie RT, Haaland ØA, Kessler J (2021). Sleep and physical activity from before conception to the end of pregnancy in healthy women: A longitudinal actigraphy study. Sleep Med..

[47] Robinson HP (1973). Sonar measurement of fetal crown-rump length as means of assessing maturity in first trimester of pregnancy. Br. Med. J..

[48] Lohman T (1988). Anthropometric Standardization Reference Manual.

[49] *Body Composition Analyser BC-418-MA. Instruction Manual* (Tanita Corporation of America. https://www.tanita.com/es/.downloads/download/?file=855638086&fl=en_US,

[50] Liden CB (2001). Benefits of the SenseWear armband over other physical activity and energy expenditure measurement techniques. White Papers Body Media.

[51] Tremblay MS (2017). Sedentary behavior research network (SBRN)—Terminology consensus project process and outcome. Int. J. Behav. Nutr. Phys. Act..

[52] Thompson, P. D. In *ACSM’ Guidelines for Exercise Testing and Prescription* (eds Pescatello, L. S., Arena, R., Riebe, D. & Thompson, P. D.) 2–14 (Lippincott Williams & Wilkins, 2014).

[53] Matthews CE, Ainsworth BE, Thompson RW, Bassett DR (2002). Sources of variance in daily physical activity levels as measured by an accelerometer. Med. Sci. Sports Exerc..

[54] Andre, D. et al. The development of the SenseWear® armband, a revolutionary energy assessment device to assess physical activity and lifestyle. *BodyMedia Inc* (2006).

[55] Bhammar DM, Sawyer BJ, Tucker WJ, Lee J-M, Gaesser GA (2016). Validity of SenseWear® Armband v5.2 and v2.2 for estimating energy expenditure. J. Sports Sci..

[56] Berntsen S, Stafne SN, Mørkved S (2011). Physical activity monitor for recording energy expenditure in pregnancy. Acta Obstet. Gynecol. Scand..

[57] Smith KM, Lanningham-Foster LM, Welk GJ, Campbell CG (2012). Validity of the SenseWear® Armband to predict energy expenditure in pregnant women. Med. Sci. Sports Exerc..

[58] Bhide A (2013). ISUOG practice guidelines: use of Doppler ultrasonography in obstetrics. Ultrasound Obstetr. Gynecol..

[59] Preisler J (2015). Defining safe criteria to diagnose miscarriage: prospective observational multicentre study. BMJ.

[60] Popović ZB, Thomas JD (2017). Assessing observer variability: a user’s guide. Cardiovasc. Diagn. Ther..

[61] Spencer TE (2014). Biological roles of uterine glands in pregnancy. Semin. Reprod. Med..

