# Supplementary material for: Maternal physical activity affects yolk sac size and growth in early pregnancy, but girls and boys use different strategies
Source: Sci Rep. 2023 Nov 20;13:20246. doi: 10.1038/s41598-023-47536-4 (PMC10661167; doi:10.1038/s41598-023-47536-4)
Supplement: Supplementary file 1 — Supplementary Information 1. [file 41598_2023_47536_MOESM1_ESM.pdf]

#******************Yolk Sac Growth-Rate by Maternal Physical Activity****************
#************************Alexander Vietheer 2023-08-30********************

# Clear environment in Rstudio
```
if(T){ rm(list=ls(all=T))
  # Install and load necessary packages in Rstudio
  pkgs <- c("here","haven", "tidyverse")
  pkgs2 <- which(!(pkgs %in% installed.packages()))
  if (length(pkgs2)>0) install.packages(pkgs[pkgs2])
  library (here)
  library (haven)
  library (tidyverse) }
```
# Read in Supplementary Dataset-1
```
if(T){
  df.w1.short <- read.csv(here("Supplementary Table S1_dataset-1.csv"), header = TRUE, sep = ",",
row.names = 1)}
```

# Stratifying dataset by fetal sex
```
if(T){ df.w1.short.girls <- df.w1.short %>% filter (childSex == "female")
df.w1.short.boys <- df.w1.short %>% filter (childSex == "male") }
```

# Yolk sac growth-rate per week (ys.growth) by per 1 hour daily physical activity before conception (pad.1)

## Not stratified by embryonic sex
```
  g.mod.p.1w <- lm (ys.growth ~ pad.1.h, data = df.w1.short, na.action = na.exclude)
  summary ( g.mod.p.1w)
  confint( g.mod.p.1w)
  AIC( g.mod.p.1w)
```
## male embryos
```
  g.mod.p.2w <- lm (ys.growth ~ pad.1.h, data = df.w1.short.boys, na.action = na.exclude)
  summary ( g.mod.p.2w)
  confint( g.mod.p.2w)
  AIC( g.mod.p.2w)
```
## female embryos
```
  g.mod.p.3w <- lm (ys.growth ~ pad.1.h, data = df.w1.short.girls, na.action = na.exclude)
  summary ( g.mod.p.3w)
  confint( g.mod.p.3w)
  AIC( g.mod.p.3w)
```
## interaction term Physical activity:embryonic sex
```
  g.mod.p.int.1.w<- lm (ys.growth ~ pad.1.h*childSex, data = df.w1.short, na.action = na.exclude)
  summary ( g.mod.p.int.1.w)
  confint( g.mod.p.int.1.w)
  AIC( g.mod.p.int.1.w)
```
# Yolk sac growth-rate per week (ys.growth) by per 1 hour daily physical activity at 13 weeks (pad.2)

## Not stratified by embryonic sex
```
  g.mod.p.4 <- lm (ys.growth ~ pad.2.h, data = df.w1.short, na.action = na.exclude)
  summary ( g.mod.p.4)
  confint( g.mod.p.4)
  AIC( g.mod.p.4)
```
## male embryos
```
  g.mod.p.5 <- lm (ys.growth ~ pad.2.h, data = df.w1.short.boys, na.action = na.exclude)
  summary ( g.mod.p.5)
  confint( g.mod.p.5)
  AIC( g.mod.p.5)
```
## female embryos
```
  g.mod.p.6 <- lm (ys.growth ~ pad.2.h, data = df.w1.short.girls, na.action = na.exclude)
  summary ( g.mod.p.6)
  confint( g.mod.p.6)
  AIC( g.mod.p.6)
```
## interaction term Physical activity:embryonic sex
```
  g.mod.p.int.2 <- lm (ys.growth ~ pad.2.h*childSex, data = df.w1.short, na.action = na.exclude)
  summary ( g.mod.p.int.2)
  confint( g.mod.p.int.2)
  AIC( g.mod.p.int.2)
```
