# Supplementary material for: Maternal physical activity affects yolk sac size and growth in early pregnancy, but girls and boys use different strategies
Source: Sci Rep. 2023 Nov 20;13:20246. doi: 10.1038/s41598-023-47536-4 (PMC10661167; doi:10.1038/s41598-023-47536-4)
Supplement: Supplementary file 2 — Supplementary Information 2. [file 41598_2023_47536_MOESM2_ESM.pdf]

Supplementary

Figure S1 Longitudinal changes of the daily physical activity duration from before pregnancy to the end of the 1st trimester (week 13). The mean difference of the activity duration was 1 h 36 min (95% CI [1 h 55 min–1 h 19 min], *p<0.001)*.

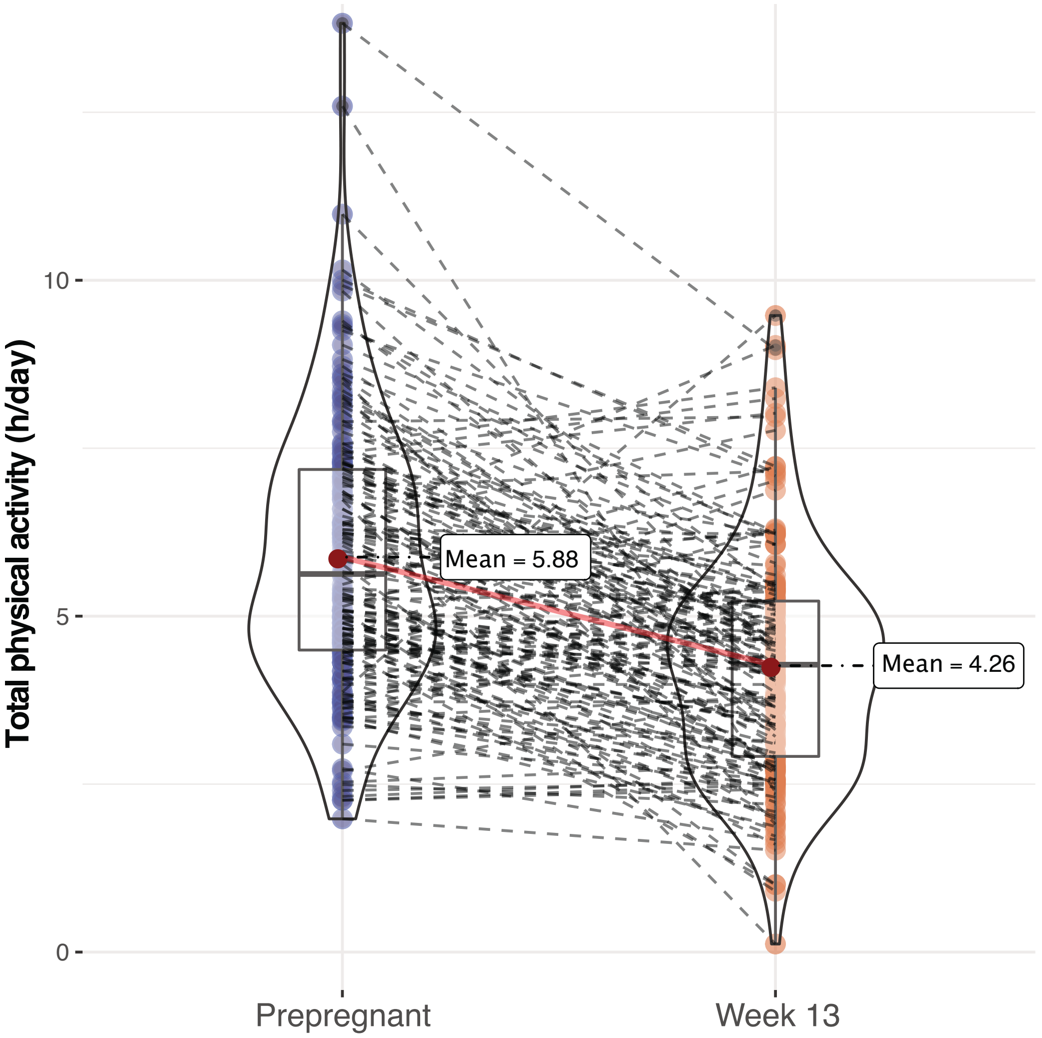

Table S1 (Dataset 1)

Please find the table in *"Supplementary Table S1_dataset-1.csv*".

Table S2 (Dataset-1 key)

Variable name	Description
id	Participant ID
inc.date_cat	Inclusion during the first, second, or third tertial of the study period
age	Maternal age before conception (at study entry)
para	Number of previous births before conception (at study entry)
ht	Maternal body height before conception (at study entry)
wt.1	Maternal body weight before conception (at study entry)
bmi.1	Maternal body mass index before conception (at study entry)
lbm.1	Maternal lean body mass before conception (at study entry)
bfp.1	Maternal body fat percent
sw.ndays.1	Number of recorded days at the first actigraphy recording (at study entry)
sw.ndays.2	Number of recorded days at the second actigraphy recording (at week 13)
pad.1.h	Total daily physical activity duration in hours at the first actigraphy recording before conception (at study entry)
pad.2.h	Total daily physical activity duration in hours at the second actigraphy recording (at week 13)
modvig_pad.1.h	Moderate and vigorous daily physical activity duration in hours at the first actigraphy recording before conception (at study entry)
modvig_pad.2.h	Moderate and vigorous daily physical activity duration in hours at the second actigraphy recording (at week 13)
lt_pad.1.h	Light daily physical activity duration in hours at the first actigraphy recording before conception (at study entry)
lt_pad.2.h	Light daily physical activity duration in hours at the second actigraphy recording (at week 13)
g_date.1	Date of the first yolk sac measurement
lmp_ga.1	Gestational age in weeks at the first yolk sac measurement at week 7 (by LMP*)
g_date.2	Date of the second yolk sac measurement
lmp_ga.2	Gestational age in weeks at the second yolk sac measurement at week 10 (by LMP)
day.diff.1	Number of days between the two yolk sac measurements
ys.1	Yolk sac size in mm at the first yolk sac measurement at week 7
ys.2	Yolk sac size in mm at the second yolk sac measurement at week 10
ys.growth	Average yolk sac growth in mm per week between the first and second measurement (week 7–10)
childSex	Sex of the child determined at birth

* LMP, the first day of the last menstrual period.

Table S3 Estimated *yolk sac diameter* at gestational week 7 (upper half) and week 10 (lower half) by total daily physical activity duration (TPAD) before pregnancy and at the end of the first trimester (week 13): Ungrouped (all); grouped according to fetal sex (male, female), or by the interaction term male sex and TPAD (Male:TPAD). Modeled using ordinary least square regression—degrees of freedom (DF); unstandardized regression coefficient (Effect); adjusted R squared (Adj.R2); 95% confidence interval (95%CI); AIC (Akaike information criterion).

Group	DF	Effect	95% CI	Adj.*r*2	AIC	*p*
**Yolk sac at week 7 by TPAD before pregnancy**						
**All**	165	0.04 mm·h^{-1}	(-0.00–0.08)	0.014	295.2	0.07
**Male**	82	0.08 mm·h^{-1}	(0.02–0.13)	0.077	137.2	**<0.01**
**Female**	81	0.00 mm·h^{-1}	(-0.06–0.07)	-0.012	158.7	0.93
**Male:TPAD**	163	0.07 mm·h^{-1}	(-0.01–0.16)	0.024	295.4	0.09
**Yolk sac at week 7 by TPAD at the end of 1st trimester**						
**All**	167	0.02 mm·h^{-1}	(-0.03–0.07)	-0.002	301.9	0.47
**Male**	83	0.04 mm·h^{-1}	(-0.04–0.11)	-0.000	146.0	0.34
**Female**	82	0.01 mm·h^{-1}	(-0.07–0.08)	-0.011	159.8	0.88

Group	DF	Effect	95% CI	Adj.*r*2	AIC	*p*
**Yolk sac at week 10 by TPAD before pregnancy**						
**All**	164	0.03 mm·h^{-1}	(-0.04–0.09)	-0.002	446.1	0.42
**Male**	81	-0.09 mm·h^{-1}	(-0.18 to -0.00)	0.038	209.8	**0.04**
**Female**	81	0.16 mm·h^{-1}	(0.06–0.26)	0.094	227.9	**<0.01**
**Male:TPAD**	162	-0.24 mm·h^{-1}	(-0.38 to -0.12)	0.064	436.7	**<0.001**
**Yolk sac at week 10 by TPAD at the end of 1st trimester**						
**All**	166	-0.01 mm·h^{-1}	(-0.10–0.07)	-0.005	451.1	0.72
**Male**	82	-0.09 mm·h^{-1}	(-0.21–0.02)	0.020	214.8	0.10
**Female**	82	0.05 mm·h^{-1}	(-0.07–0.17)	-0.003	238.1	0.41

Table S4 Estimated *male yolk sac diameter* at gestational *week 7* by total daily activity duration (TPAD) *before pregnancy*. Calculated by ordinary least square regression models—unstandardized regression coefficient (Effect); adjusted R squared (Adj.R2); 95% confidence interval (95%CI); AIC (Akaike information criterion). The crude model (grey background) with its estimates and significance level can be compared with the adjusted models listened below, controlling for maternal health parameters: i.e., age, parity, height, weight, BMI, lean body mass (LBM), body fat percent (BFP), gestational age (GA), and stratified date of inclusion (inc. date; 3 time-categories). The last row represents the crude model but based on women without pregnancy complications (compl. excl.). The activity-yolk sac relation remained significant after adjustments that caused minute changes in the estimated effect.

Model	DF	Effect	95% CI	Adj.*r*2	AIC	*p*
**Crude**	82	0.07 mm·h^{-1}	(0.02–0.13)	0.077	137.2	**<0.01**
**Adjusted by age**	81	0.08 mm·h^{-1}	(0.03–0.14)	0.085	137.0	**<0.01**
**Adjusted by parity**	79	0.07 mm·h^{-1}	(0.01–0.13)	0.062	141.0	**0.02**
**Adjusted by height**	81	0.07 mm·h^{-1}	(0.01–0.13)	0.085	136.9	**0.02**
**Adjusted by weight**	81	0.09 mm·h^{-1}	(0.03–0.14)	0.099	135.6	**<0.01**
**Adjusted by BMI**	81	0.09 mm·h^{-1}	(0.03–0.15)	0.071	138.3	**<0.01**
**Adjusted by LBM**	81	0.08 mm·h^{-1}	(0.02–0.13)	0.114	134.2	**<0.01**
**Adjusted by BFP**	81	0.08 mm·h^{-1}	(0.02–0.14)	0.065	138.7	**<0.01**
**Adjusted by GA**	81	0.07 mm·h^{-1}	(0.02–0.13)	0.118	133.9	**0.01**
**Adjusted by incl. date**	80	0.08 mm·h^{-1}	(0.02–0.14)	0.059	140.2	**<0.01**
**Crude compl. excl.**	70	0.06 mm·h^{-1}	(0.00–0.12)	0.045	111.2	**0.04**

Table S5 Estimated *female yolk sac diameter* at gestational *week 7* by total daily activity duration *(TPAD) before pregnancy*. Calculated by ordinary least square regression models—unstandardized regression coefficient (Effect); adjusted R squared (Adj.R2); 95% confidence interval (95%CI); AIC (Akaike information criterion). The crude model (grey background) with its estimates and significance level can be compared with the adjusted models listened below, controlling for maternal health parameters: i.e., age, parity, height, weight, BMI, lean body mass (LBM), body fat percent (BFP), gestational age (GA), and stratified date of inclusion (inc. date; 3 time-categories). The last row represents the crude model but based on women without pregnancy complications (compl. excl.). The activity-yolk sac relation remained constant after adjustments that caused minute changes in the estimated effect.

Model	DF	Effect	95% CI	Adj.$r2$	AIC	p
**Crude**	81	0.00 $mm \cdot h^{-1}$	(-0.06–0.07)	-0.012	158.7	0.93
**Adjusted by age**	80	-0.00 $mm \cdot h^{-1}$	(-0.07–0.07)	0.020	160.3	>0.99
**Adjusted by parity**	79	-0.00 $mm \cdot h^{-1}$	(-0.07–0.07)	-0.035	162.4	>0.99
**Adjusted by height**	80	-0.00 $mm \cdot h^{-1}$	(-0.07–0.07)	-0.020	160.3	0.96
**Adjusted by weight**	80	-0.01 $mm \cdot h^{-1}$	(-0.08–0.06)	-0.012	159.6	0.81
**Adjusted by BMI**	80	-0.01 $mm \cdot h^{-1}$	(-0.08–0.07)	-0.018	160.1	0.87
**Adjusted by LBM**	80	-0.00 $mm \cdot h^{-1}$	(-0.07–0.07)	-0.022	160.4	>0.99
**Adjusted by BFP**	80	-0.01 $mm \cdot h^{-1}$	(-0.08–0.06)	-0.010	159.5	0.77
**Adjusted by GA**	80	0.01 $mm \cdot h^{-1}$	(-0.06–0.07)	0.060	153.4	0.86
**Adjusted by incl. date**	79	0.00 $mm \cdot h^{-1}$	(-0.06–0.07)	-0.002	159.7	0.90
**Crude compl. excl.**	75	0.00 $mm \cdot h^{-1}$	(-0.08–0.07)	-0.013	147.3	0.94

Table S6 Estimated *male yolk sac diameter* at gestational *week 10* by total daily activity duration *(TPAD) before pregnancy*. Calculated by ordinary least square regression models—unstandardized regression coefficient (Effect); adjusted R squared (Adj.R2); 95% confidence interval (95%CI); AIC (Akaike information criterion). The crude model (grey background) with its estimates and significance level can be compared with the adjusted models listened below, controlling for maternal health parameters: i.e., age, parity, height, weight, BMI, lean body mass (LBM), body fat percent (BFP), and stratified date of inclusion (inc. date; 3 time-categories). The last row represents the crude model but based on women without pregnancy complications (compl. excl.). The activity-yolk sac relation remained significant after adjustments that caused minute changes in the estimated effect.

Model	DF	Effect	95% CI	Adj.*r2*	AIC	*p*
**Crude**	81	-0.09 mm·h^{-1}	(-0.18 to -0.00)	0.038	209.8	**0.04**
**Adjusted by age**	80	-0.09 mm·h^{-1}	(-0.18–0.00)	0.023	211.6	0.06
**Adjusted by parity**	78	-0.09 mm·h^{-1}	(-0.18–0.00)	0.027	213.7	0.06
**Adjusted by height**	80	-0.09 mm·h^{-1}	(-0.18 to -0.00)	0.026	211.8	**0.04**
**Adjusted by weight**	80	-0.08 mm·h^{-1}	(-0.17–0.01)	0.036	211.0	0.07
**Adjusted by BMI**	80	-0.08 mm·h^{-1}	(-0.04–0.02)	0.037	210.9	0.10
**Adjusted by LBM**	80	-0.09 mm·h^{-1}	(-0.18 to -0.00)	0.032	211.4	**0.04**
**Adjusted by BFP**	80	-0.08 mm·h^{-1}	(-0.17–0.01)	0.034	211.2	0.09
**Adjusted by GA**	80	-0.09 mm·h^{-1}	(-0.18 to -0.00)	0.026	211.8	**0.04**
**Adjusted by incl. date**	79	-0.09 mm·h^{-1}	(-0.18–0.00)	0.021	213.2	**0.04**
**Crude compl. excl.**	69	-0.08 mm·h^{-1}	(-0.18–0.01)	0.027	181.2	0.09

Table S7 Estimated the *female yolk sac diameter* at gestational *week 10* by total daily activity duration *(TPAD) before pregnancy*. Calculated by ordinary least square regression models—unstandardized regression coefficient (Effect); adjusted R squared (Adj.R2); 95% confidence interval (95%CI); AIC (Akaike information criterion). The crude model (grey background) with its estimates and significance level can be compared with the adjusted models listened below, controlling for maternal health parameters: i.e., age, parity, height, weight, BMI, lean body mass (LBM), body fat percent (BFP), and stratified date of inclusion (inc. date; 3 time-categories). The last row represents the crude model but based on women without pregnancy complications (compl. excl.). The activity-yolk sac relation remained significant after adjustments that caused minute changes in the estimated effect.

Model	DF	Effect	95% CI	Adj.*r2*	AIC	*p*
**Crude**	81	0.16 $mm \cdot h^{-1}$	(0.06–0.26)	0.094	227.9	**<0.01**
**Adjusted by age**	80	0.15 $mm \cdot h^{-1}$	(0.05–0.25)	0.093	229.0	**<0.01**
**Adjusted by parity**	79	0.14 $mm \cdot h^{-1}$	(0.04–0.24)	0.108	228.5	**<0.01**
**Adjusted by height**	80	0.16 $mm \cdot h^{-1}$	(0.06–0.26)	0.083	229.9	**<0.01**
**Adjusted by weight**	80	0.15 $mm \cdot h^{-1}$	(0.04–0.26)	0.087	229.5	**<0.01**
**Adjusted by BMI**	80	0.14 $mm \cdot h^{-1}$	(0.03–0.25)	0.090	229.3	**0.01**
**Adjusted by LBM**	80	0.16 $mm \cdot h^{-1}$	(0.05–0.26)	0.083	229.9	**<0.01**
**Adjusted by BFP**	80	0.13 $mm \cdot h^{-1}$	(0.02–0.24)	0.099	228.4	**0.01**
**Adjusted by GA**	80	0.17 $mm \cdot h^{-1}$	(0.07–0.27)	0.204	212.4	**<0.01**
**Adjusted by incl. date**	79	0.16 $mm \cdot h^{-1}$	(0.06–0.27)	0.074	231.6	**<0.01**
**Crude compl. excl.**	75	0.17 $mm \cdot h^{-1}$	(0.06–0.28)	0.099	211.2	**<0.01**

Table S8 Estimated yolk sac Z-score at gestational week 7 (upper half) and week 10 (lower half) by total daily physical activity duration (TPAD) before pregnancy and at the end of the first trimester (week 13): Grouped according to fetal sex (male, female). Modeled using ordinary least square regression—degrees of freedom (DF); unstandardized regression coefficient (Effect); adjusted R squared (Adj.R2); 95% confidence interval (95%CI); AIC (Akaike information criterion).

Group	DF	Effect	95% CI	Adj.$r2$	AIC	p
**Yolk sac Z-score at week 7 by daily PAD before pregnancy**						
**Male**	80	$0.20{\cdot}h^{-1}$	(0.07–0.33)	0.09	270.4	**<0.01**
**Female**	78	$-0.03{\cdot}h^{-1}$	(-0.19–0.12)	-0.01	278.7	0.65
**Yolk sac Z-score at week 7 by daily PAD at the end of 1st trimester**						
**Male**	80	$0.06{\cdot}h^{-1}$	(-0.11–0.23)	-0.006	278.8	0.49
**Female**	78	$0.02{\cdot}h^{-1}$	(-0.14–0.19)	-0.012	278.8	0.80
**Yolk sac Z-score at week 10 by daily PAD before pregnancy**						
**Male**	79	$-0.14{\cdot}h^{-1}$	(-0.26 to -0.01)	0.044	263.8	**0.03**
**Female**	78	$0.21{\cdot}h^{-1}$	(0.06–0.35)	0.085	270.7	**<0.01**
**Yolk sac Z-score at week 10 by daily PAD at the end of 1st trimester**						
**Male**	79	$-0.12{\cdot}h^{-1}$	(-0.28–0.05)	0.012	266.4	0.16
**Female**	78	$0.03{\cdot}h^{-1}$	(-0.13–0.20)	-0.011	278.7	0.69

Equations Eq.1 Z-score calculation:

In this section, we will explain how Z-scores were obtained, and how means and standard deviations (SDs) were estimated for each of the measurements. First, we modelled the mean yolk sac size for individual *j* at time *i* using a 2nd order polynomial mixed model with random intercept,

$$\mu_{ij} = \beta 0_j + \beta 1_j \times GA_{ij} + \beta 2_j \times GA_{ij}^2 + \epsilon_{ij} \ , \qquad (S1)$$

where GA is gestational age and ϵ_{ij} is a normally distributed error term. From *(S1)* we now get

$$\hat{\mu}_{ij} = b0_j + b1_j \times GA_{ij} + b2_j \times GA_{ij}^2 \ , \qquad (S2)$$

where the *b*'s are the estimated β's from *(S1)*. Because the SD will change with GA we also calculate an SD for each GA. Letting $c.res = \sqrt{\left(obs_{ij} - \hat{\mu}_{ij}\right)^2}$, where obs_{ij} are the observed yolk sac sizes. For individual *j* at time *i*, we get

$$c.res_{ij} = \alpha_0 + \alpha_1 \times GA_{ij} + \tau_{ij} \ , \qquad (S3)$$

where τ_{ij} is a normally distributed error term. Now, our modelled SD for individual *j* at time *i* becomes

$$SD_{ij} = a_0 + a_1 \times GA_{ij} \ , \qquad (S4)$$

where the a's are the estimated α's from *(S3)*. Finally, from *(S2)* and *(S4)*, we now get that the Z-score for individual *j* at time *i* is

$$Z_{ij} = \frac{obs_{ij} - \hat{\mu}_{ij}}{SD_{ij}} \ .$$

Code C1 Syntax (r-code) Z-score calculation:*)*

```
# Load necessary packages and libraries
if(T){
  rm(list=ls(all=T))
  pkgs <- c("here", "lme4")
  pkgs2 <- which(!(pkgs %in% installed.packages()))
  if (length(pkgs2)>0) install.packages(pkgs[pkgs2])
library(here)
library(lme4)
}
# Import Supplementary Table S1 (Dataset-A)
df.w1.short.ys <- read.csv(here("Supplementary Table S1_dataset-1.csv"), header =
TRUE, sep = ",", row.names = 1)
# longformat the dataset
df.l1.short.ys <- reshape(df.w1.ys.short, direction ="long",
                           varying= c("ys.1","ys.2","lmp_ga.1","lmp_ga.2"),
                           idvar="id",
                           sep=".")
# reorder by id
df.l1.short.ys <- df.l1.short.ys[order(df.l1.short.ys$id,df.l1.short.ys$time),]
df.l1.short.ys <- df.l1.short.ys %>% rename("Time of measurement"=time)
df.l1.short.ys$"Time of measurement"<- recode_factor (df.l1.short.ys$"Time of
measurement", "1"="Week 7", "2"="Week 10")

## statistical model (yolk sac by gestational age; quadratic linear random intercept
model: LME4)
Model.1 <- lmer(ys ~ lmp_ga + I(lmp_ga^2) + (1 |id), na.action = na.exclude, data
= df.l1.short.ys , REML = FALSE)

## column of predicted values
df.l1.short.ys$ys_predicted <- NA
tmp <- predict(Model.1)
## adding predicted model values to the correct individuals
df.l1.short.ys[names(tmp),"ys_predicted"] <- tmp

### Create new yolk sac standard deviation “sd-variable” with only NA
df.l1.short.ys$c.res <- NA
```

```
### Create temporary vector based and the calculated standard deviation from the residuals (converted residuals “c.res” from Model.1
c.res <- sqrt (residuals(Model.1)^2)

### Adding converted residuals of the model.1 (c.res) to the correct individuals
df.l1.short.ys[names(c.res),"c.res"] <- c.res
#### Model the converted residuals (c.res) values
Model.2 <- lm(c.res ~ lmp_ga, data = df.l1.short.ys, na.action = na.exclude)

#### get the predicted standard deviations (sd_pred) and create new variable with only NA
df.l1.short.ys$sd_pred <- NA
#### Create temporary vector from predicted standard deviations
tmp.sd.pred <- predict(Model.2)
#### Adding predicted yolk sac standard deviations to the correct individuals
df.l1.short.ys[names(tmp.sd.pred),"sd_pred"] <- tmp.sd.pred

##### Calculate the final yolk sac Z-scores based gestational age adjusted predicted mean and gestational age adjusted predicted standard deviation
##### Create new variable with only NA
df.l1.short.ys$ys_Zscore <- NA

##### Create temporary vector with the calculated Z-scores
tmp.Zscore <- (df.l1.short.ys$ys-
df.l1.short.ys$ys_predicted)/df.l1.short.ys$sd_pred
####### Adding yolk sac Z-scores to the correct individuals
df.l1.short.ys$ys_Zscore <- tmp.Zscore
```

Figure S2 Male and female yolk sac diameter at week 7 (left, *N*=180) and week 10 (right, *N*=178) by the total daily physical activity duration (TPAD) before pregnancy. Prediction lines are calculated by sex-stratified quantile regression models—median (thick black); 5th, 20th, 30th, 40th, 60th, 70th, 80th, and 95th percentile (grey); ordinary least square regression-line (stippled).

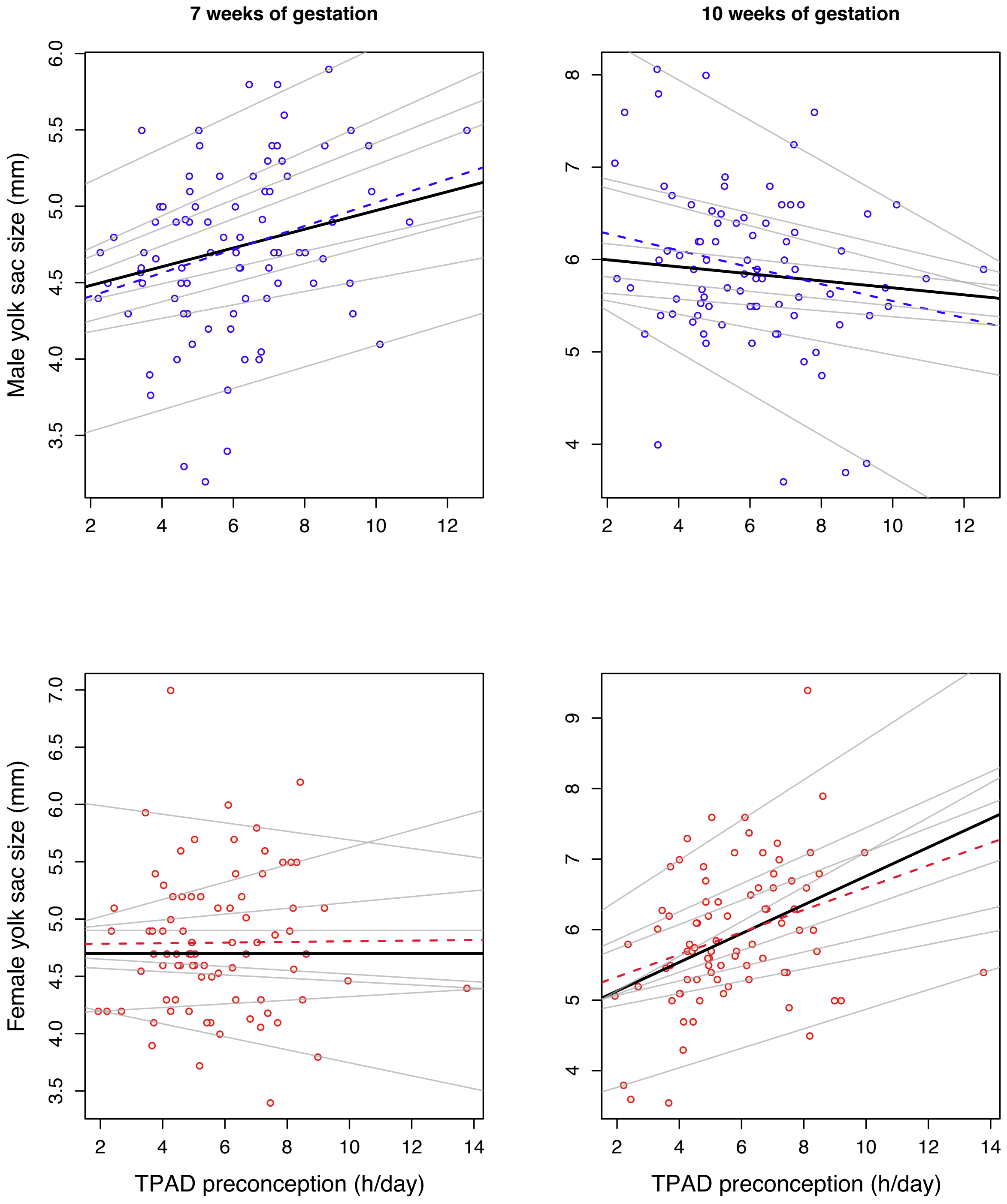

Figure S3 Overview of the sex- and time-stratified results of the quantile regression models: The **preconception** activity estimates on the y-axis with 95% confidence intervals (change of yolk sac size in mm per h of daily physical activity duration (TPAD) before conception) were calculated for yolk sac deciles on the x-axis; zero-effect (red line)

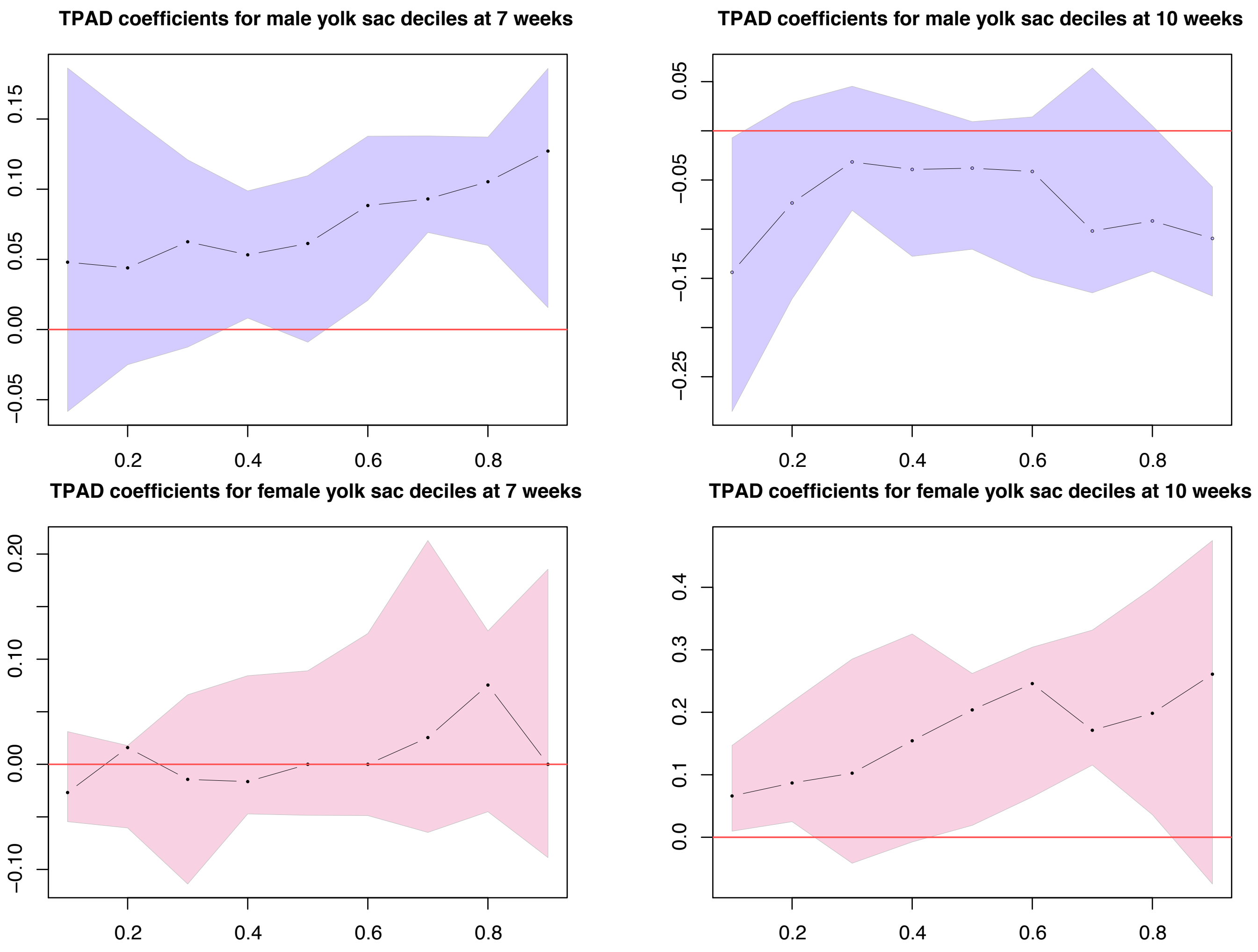

Figure S4 Unadjusted quantile regression-lines for the sex specific yolk sac measurements from the first (week 7, *N*= 180) and second measurement (week 10, *N* = 178) by daily physical activity duration at the end of the 1st trimester (week 13); median (thick black line) and 5th, 20th, 30th, 40th, 60th, 70th, 80th, and 95th percentile (grey); Individual observations (open circles); ordinary least square regression-line (stippled).

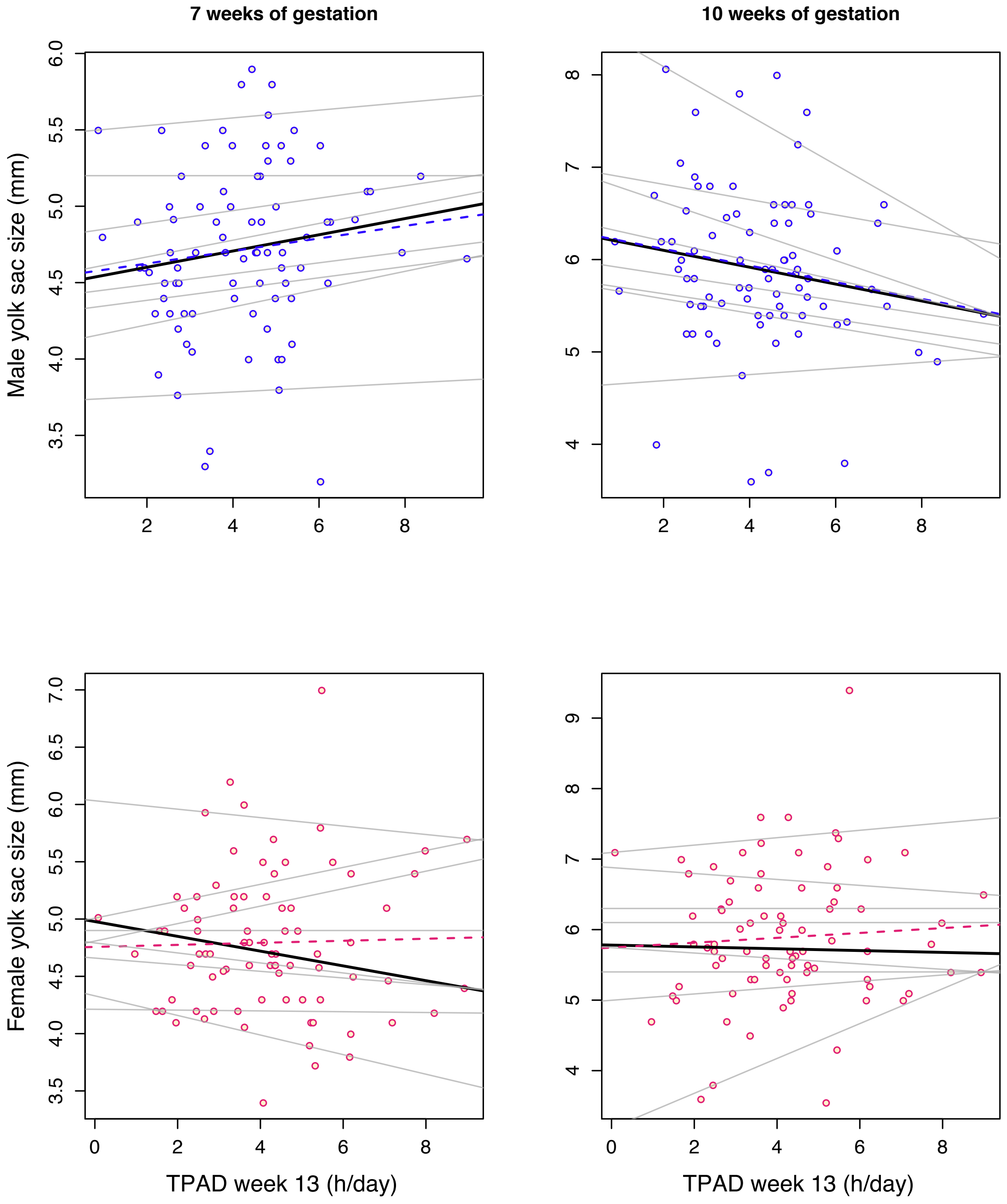

Figure S5 Overview of the sex- and time-stratified results of the quantile regression models: The **end of 1st trimester (week 13)** activity estimates on the y-axis with 95% confidence intervals (change of yolk sac size in mm per h of daily physical activity duration (TPAD) before conception) were calculated for yolk sac deciles on the x-axis; zero-effect (red line)

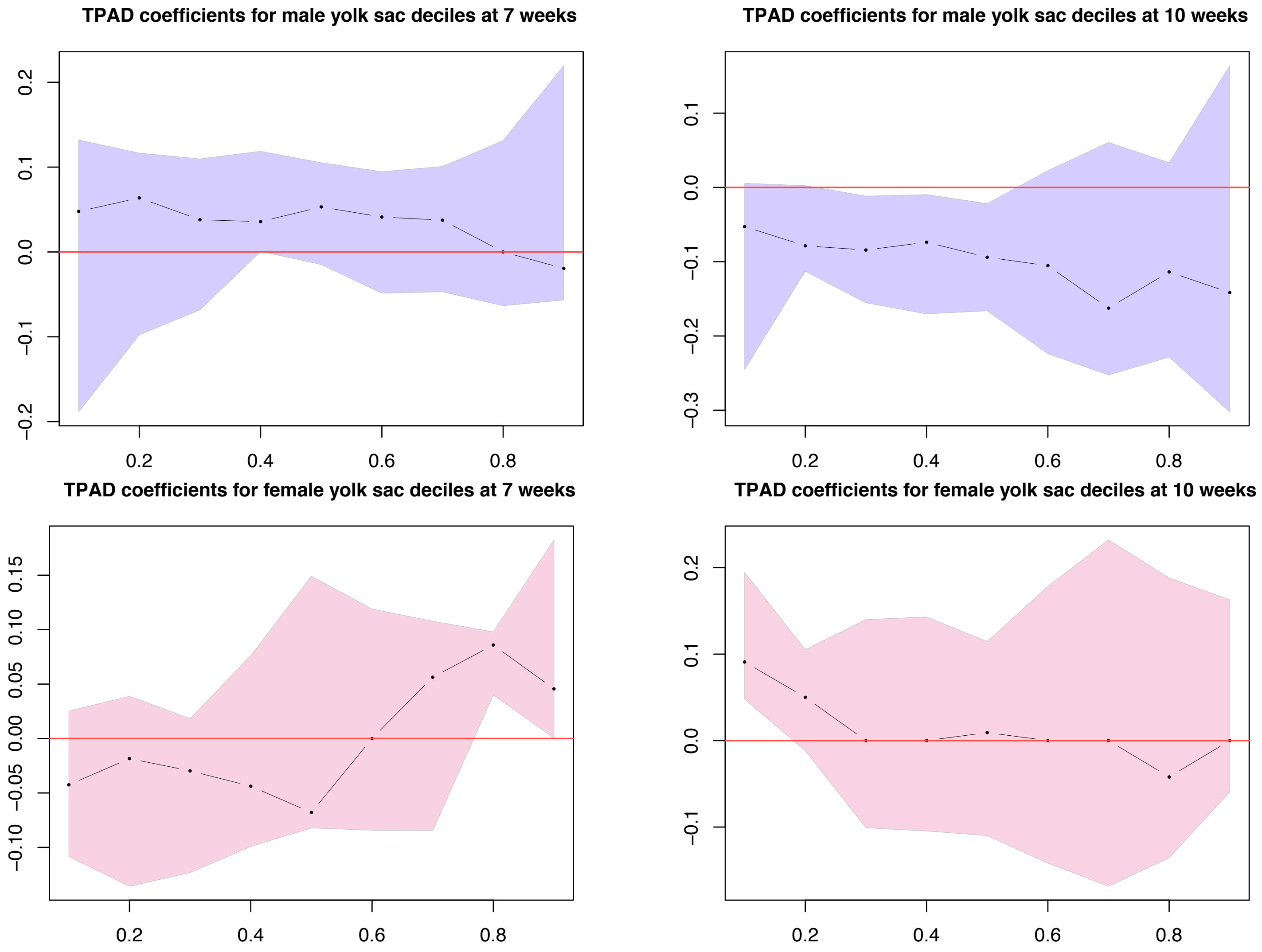

Table S9 Estimated *Yolk sac growth rate (mm·week^{-1}·h^{-1})* by total daily physical activity duration (TPAD) before pregnancy and at the end of the first trimester (week 13): Ungrouped (all); grouped according to fetal sex (male, female), or by the interaction term male sex and TPAD (Male:TPAD). Modeled using ordinary least square regression—degrees of freedom (DF); unstandardized regression coefficient (Effect); adjusted R squared (Adj.R2); 95% confidence interval (95%CI); AIC (Akaike information criterion).

Model	DF	Effect	95% CI	Adj.*r*2	AIC	*p*
**Yolk sac growth rate by daily PAD before pregnancy**						
**All**	156	-0.00 mm·week^{-1}·h^{-1}	(-0.03–0.02)	-0.006	103.3	0.77
**Male**	75	-0.05 mm·week^{-1}·h^{-1}	(-0.08 to -0.02)	0.126	26.4	**<0.01**
**Female**	79	0.05 mm·week^{-1}·h^{-1}	(0.01–0.09)	0.064	62.4	**0.01**
**Male:PAD**	154	-0.1 mm·week^{-1}·h^{-1}	(-0.15 to -0.05)	0.084	90.5	**<0.01**
**Yolk sac growth rate by daily PAD at the end of first trimester**						
**All**	158	-0.01 mm·week^{-1}·h^{-1}	(-0.04–0.02)	-0.004	96.2	0.53
**Male**	76	-0.03 mm·week^{-1}·h^{-1}	(-0.07–0.01)	0.021	34.6	0.11
**Female**	80	0.01 mm·week^{-1}·h^{-1}	(-0.03–0.05)	-0.010	63.3	0.66
**Male:PAD**	156	-0.04 mm·week^{-1}·h^{-1}	(-0.10–0.02)	-0.003	98.1	0.15

Please find the code for corresponding statistics as supplementary code in:
"*R-Code-growth-rate-stats_2023-08-30.pdf*"

Table S10 Dataset 2

Please find the table in the file *“Supplementary Dataset 2”*.

Table S11 Key dataset-2

Variable name	Description
id	Subject number; 1–19 .
measurement_nr	Measurement groups; 1–2 .
Observer	Observer (Ultrasound operator; 1–7)
mean_all	Value of the yolk sac measurement in mm.

Code C2 Syntax (r-code) for dataset-2:
(Analysis of the intra- and inter-observer variation, and determination of the standard error of measurements (SEM's).

# Load necessary packages and libraries

```
if(T){rm(list=ls(all=T))
pkgs <- c("here")
pkgs2 <- which(!(pkgs %in% installed.packages()))
if (length(pkgs2)>0) install.packages(pkgs[pkgs2])
}
library(here)
```

# Import Dataset-2

```
AOV_data <- read.csv(here("Supplementary Table S10_dataset-2.csv "), header = TRUE,
sep = ",", row.names = 1))
```

# id (index for the study subjects), Observer (index for the observers), measurement_nr (index for the measurement at week 7 or 10, and mean_all is the measured value

```
attach(AOV_data)
```

# Fitting the two-way ANOVA

```
(fit<-aov(mean_all~1+factor(Observer)+factor(id)+factor(Observer)*factor(id)))
```

# Getting the fitted output of the model

```
(fita<-anova(fit))
```

# Output of the residual variation corresponds to intra-observer variation (repeatability)

```
(intra.obsvar<-fita$`Mean Sq`[4])
```

# Calculation observer variation (reproducibility)

```
(observervar<-  (fita$`Mean Sq`[1]-fita$`Mean Sq`[3])/(length(unique(id))*length
                (unique(exam_nr))))
```

# Calculation interaction variation

```
(interactionvar<-(fita$`Mean Sq`[3]-fita$`Mean Sq`[4])/length(unique(exam_nr)))
```

# Inter-observer variation

```
(inter.obsvar <- intra.obsvar+observervar+interactionvar)
```

# Calculation SEM of the intra-observer variation

```
(SEMintra<-sqrt(intra.obsvar))
```

# SEM of the inter-observer variation fixed effect

```
(SEMinter.fixed<-sqrt(observervar))
```

# SEM's of the inter observer variation random effect

```
(SEMinter.random<-sqrt(inter.obsvar))
detach(AOV_data)
```
